# The Small Ones Matter—sHsps in the Bacterial Chaperone Network

**DOI:** 10.3389/fmolb.2021.666893

**Published:** 2021-05-13

**Authors:** Igor Obuchowski, Piotr Karaś, Krzysztof Liberek

**Affiliations:** Intercollegiate Faculty of Biotechnology UG-MUG, University of Gdansk, Gdansk, Poland

**Keywords:** protein aggregation, protein refolding, holdase activity, chaperones, small heat shock proteins (sHsps), proteotoxic stress, Hsp70–Hsp100 dependent disaggregation

## Abstract

Small heat shock proteins (sHsps) are an evolutionarily conserved class of ATP-independent chaperones that form the first line of defence during proteotoxic stress. sHsps are defined not only by their relatively low molecular weight, but also by the presence of a conserved α-crystallin domain, which is flanked by less conserved, mostly unstructured, N- and C-terminal domains. sHsps form oligomers of different sizes which deoligomerize upon stress conditions into smaller active forms. Activated sHsps bind to aggregation-prone protein substrates to form assemblies that keep substrates from irreversible aggregation. Formation of these assemblies facilitates subsequent Hsp70 and Hsp100 chaperone-dependent disaggregation and substrate refolding into native species. This mini review discusses what is known about the role and place of bacterial sHsps in the chaperone network.

## Introduction

Bacterial sHsps, unlike most other chaperones, were discovered later than their eukaryotic homologues. They were originally found in *Escherichia coli* inclusion bodies (Allen et al., 1992), hence they were given names IbpA and IbpB inclusion body-associated protein A and B. They were later reported to interact with endogenous polypeptides upon heat stress conditions and therefore classified as members of the chaperone family (Laskowska et al., 1996).

The level of sHsps in bacteria is very low at physiological conditions. This is due to very tight regulation of sHsp expression at both transcriptional and translational levels. In *E. coli ibpA* and *ibpB* genes are arranged into an operon which is controlled by σ^32^, the main heat shock response regulator (Allen et al., 1992; Chuang and Blattner 1993; Kuczyńska-Wisńik et al., 2001). The deletion of the *ibpAB* operon does not influence *E. coli* growth in permissive conditions, however during prolonged harsh stress it substantially decreases bacterial viability (Kuczynska-Wisnik et al., 2002).

After transcription at a permissive temperature, the *ibpAB* mRNA forms a hairpin structure, which restricts access to its own Shine-Dalgarno sequence (SD) (Waldminghaus et al., 2009; Gaubig et al., 2011), preventing unnecessary translation. Additionally, oligomeric IbpA negatively regulates its own translation by directly binding to *ibpAB* mRNA, which promotes the mRNA degradation by polynucleotide phosphorylase (Miwa et al., 2021). At the protein level, excessive sHsps are effectively degraded by Lon protease (Bissonnette et al., 2010).

At stress conditions the expression of sHsps rapidly increases. This is orchestrated by the σ^32^ transcription activation, meltdown of the SD-covering mRNA hairpin structure (Waldminghaus et al., 2009) and heat-induced deoligomerization and dissociation of IbpA from its own mRNA (no more degradation stimulation) (Miwa et al., 2021). This, in *E. coli*, causes ∼300 fold induction of the sHsp expression at the transcriptional level (Richmond et al., 1999), which results in a very dynamic 20-fold increase in the cellular abundance of sHsps (Valdez-Cruz et al., 2011; Laskowska et al., 1996; Mogk et al., 1999). This is in contrast to other heat shock proteins, whose cellular levels typically increase only 2–3 times in similar conditions (Mogk et al., 1999).

Such unusually tight multilevel control of IbpA and IbpB expression in *E. coli* points to their importance at stress conditions and suggests that at physiological conditions sHsps may exert some negative effects on bacterial growth. Indeed, it was recently observed that the overexpression of IbpA inhibits *E. coli* growth (Miwa et al., 2021). It was also observed that the expression of *Mycobacterium tuberculosis* sHsp16.3 arrests cell growth, which in the case of TB is beneficial, as it allows the bacteria to establish the characteristic latent infection (Hu et al., 2006).

Although bacterial sHsp expression studies explored mostly *E. coli*, less investigated bacterial systems seem to generally show similar trends of the heat-dependent sHsps expression. Analyzing sHsp genes from multiple alpha- and gamma-proteobacteria, Narberhaus and colleagues have shown that, similarly to *E. coli* sHsps, they possess RNA thermometers within SD sequences (Narberhaus et al., 2006) that form hairpins on the mRNA structure and melt upon a temperature rise to promote the translation initiation.

## Structure of Bacterial Small Heat Shock Proteins

The secondary and tertiary structure of bacterial sHsps is highly conserved. The central ∼90 aa α-crystallin domain is the basic structural element which defines the membership in the sHsp family (Haslbeck and Vierling 2015; Basha et al., 2013). The α-crystallin domain consists of two antiparallel ß-sheets, formed by three and four β-strands, as well as an extended, so-called dimerization loop. This structure is conserved among bacterial and other, non-metazoan sHsps (Hilario et al., 2011; Mani et al., 2016). The α-crystallin domain is flanked by highly divergent, partially unstructured, flexible N- and C - terminal extensions. These tend to be enriched in prolines, which may contribute to the reduced amount of secondary structures present in these termini (Kriehuber et al., 2010). A highly conserved feature of the C-terminal extension is the (I/V)-X-(I/V) motif (Haslbeck and Vierling 2015), preceded by a positively charged amino acid (in *E. coli* IbpA - arginine 133) (Strozecka et al., 2012).

A characteristic feature of all sHsps is their ability to form oligomers. Known structures of bacterial sHsp oligomers include tetrahedral 12-mers formed by *M. tuberculosis* Hsp 16.3 (Kennaway et al., 2005), as well as the 18-meric trigonal bipyramid and the 24-meric octahedron formed by *Salmonella typhimurium* AgsA (Mani et al., 2016). Deinococcus *radiodurans* Hsp 20.2 is able to form 18-mers and 36-mers (Bepperling et al., 2012) and *E. coli* IbpA and IbpB form large, polydisperse oligomers up to several MDa in size (Shearstone and Baneyx 1999; Matuszewska et al., 2005), IbpA also being able to form fibrils *in vitro* in the absence of IbpB (Ratajczak et al., 2010).

The oligomers are formed by sHsp dimers that interact with each other and build higher-order structures (Kennaway et al., 2005; Hilario et al., 2011; Mani et al., 2016). A notable exception is Hsp 17.7 from D. ra*diodurans* that does not form higher-order oligomers and exists exclusively as a dimer (Bepperling et al., 2012). Interactions between α-crystallin domains play a crucial role in the formation of the dimers. In the case of bacterial sHsps, the dimer is stabilized mainly by interactions between the extended loop on one monomer and two β- strands on the other monomer (Hilario et al., 2011; Bepperling et al., 2012; Mani et al., 2016).

While not required for sHsp dimerization, the N- and C- terminal extensions play a crucial role in the formation of higher-order oligomers (Mani et al., 2016; Strozecka et al., 2012; Bepperling et al., 2012; Fu et al., 2005). The conserved C-terminal (I/V)-X-(I/V) motif interacts with a hydrophobic groove formed by two β-sheets on the α-crystallin domain of the other sHsp, providing an anchoring interaction between adjacent dimeric units in the sHsp oligomer (Kennaway et al., 2005; Bepperling et al., 2012). The N-terminal extensions tend to group together inside the oligomer structure and their deletion prevents the formation of higher order oligomers (Kennaway et al., 2005). Current understanding of the N-terminal extension detailed role in bacterial sHsp oligomerization is limited by the difficulty in obtaining high-quality crystallographic data, likely due to N-terminus mobility in the oligomer (Hilario et al., 2011; Mani et al., 2016).

## Activities of Bacterial Small Heat Shock Proteins

Since eukaryotic (also human) sHsps were discovered before their bacterial homologues, the majority of biochemical data describing sHsp activities come from eukaryotic systems studies. It is somehow anticipated that bacterial sHsps possess similar biochemical properties since sHsps from both groups are structurally similar (Haslbeck et al., 2019). Both eukaryotic (Friedrich et al., 2004; Painter et al., 2008; Benesch et al., 2010) and bacterial sHsps oligomer populations (Shearstone and Baneyx 1999; Jiao et al., 2005) are in dynamic equilibrium and upon temperature raise tend to shift toward smaller species. Oligomerized sHsps are considered an inactive, storage form of sHsps and it is the heat-dissociated smaller species (dimers?), that are believed to be responsible for their chaperone activity (Haslbeck and Vierling 2015).

**FIGURE 1:**
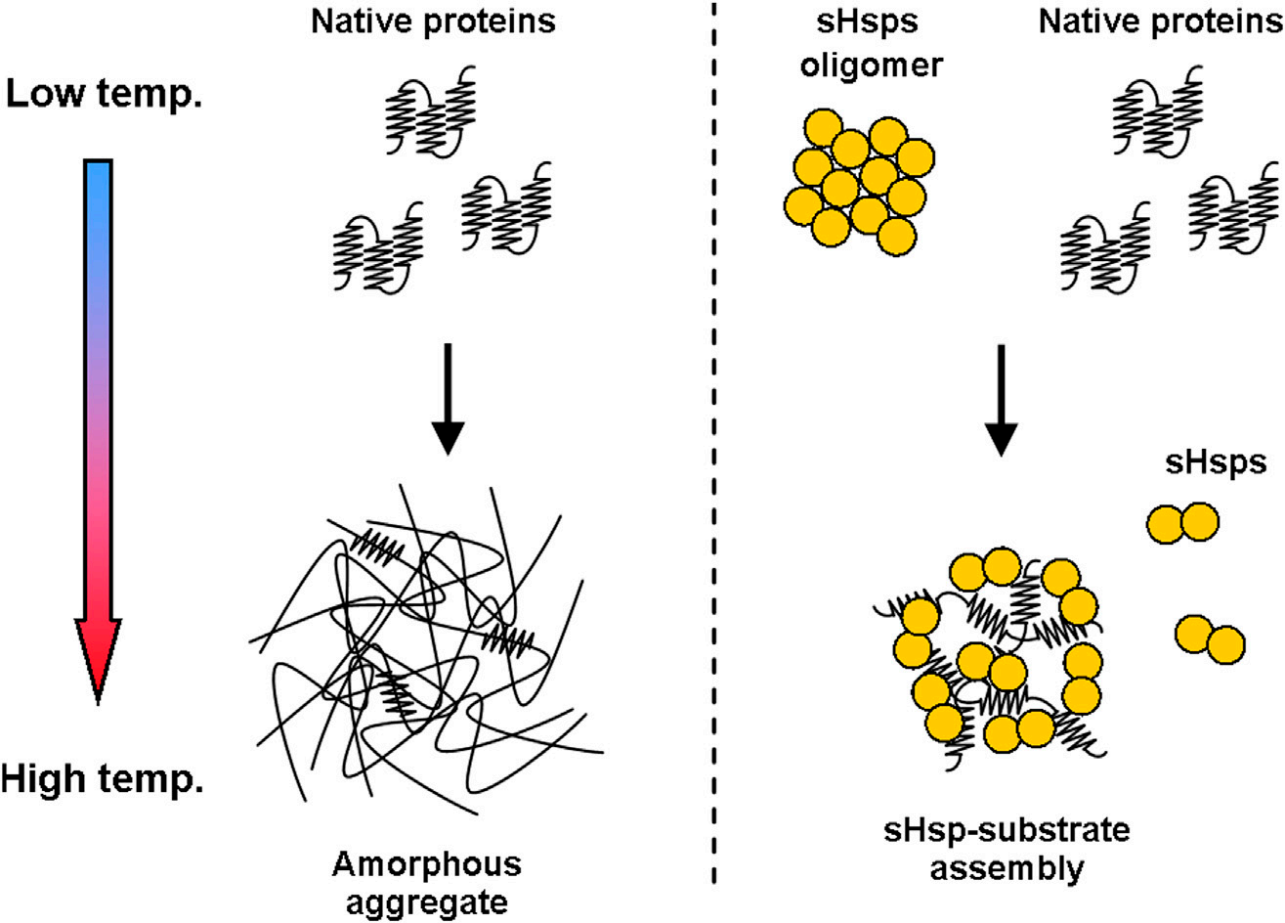
sHsps influence the substrate aggregation process. Temperature increase causes deoligomerization and activation of sHsps, which start binding to partially unfolded polypeptide substrates. This modifies the aggregation process and leads to the formation of sHsp-substrate assemblies.

The canonical chaperone activity of sHsps is ATP-independent and is based on scavenging unfolding polypeptides before they spontaneously aggregate and either quickly releasing them after a swift stabilization or more permanently complexing them into so-called sHsp-substrate assemblies (Figure 1)—at least *in vitro* (Haslbeck et al., 2005). It is still unclear what is the discriminating factor that drives the process towards either the first or the second path, it is however speculated to rely on the unfolding state/hydrophobicity of the substrate.

The quick bind-and-release activity path of bacterial sHsps can be observed both *in vitro* and *in vivo* in enzyme activity protection assays. In this way *E. coli* sHsps were shown to protect different enzymes from thermal (Fu et al., 2013; Matuszewska et al., 2005), oxidative and freeze-thaw (Kitagawa et al., 2002) inactivation. On the other hand, however, there are sHsps that are completely ineffective in this mode of activity. In turn, they are capable of stably binding polypeptides and driving their aggregation towards small assemblies (Chang et al., 1996). In fact, there are species like *D. radiodurans* that possess two different non-interacting sHsps, where each seems to be dedicated to either transient or stable interactions with unfolding polypeptides (Bepperling et al., 2012). This is in contrast to *E. coli*, where both expressed sHsps can, to some extent, protect enzymes from inactivation (Kitagawa et al., 2002; Matuszewska et al., 2005) and cooperate in stable substrate binding and disaggregation (Matuszewska et al., 2005; Ratajczak et al., 2009; Zwirowski et al., 2017). Therefore, it is not only substrate hydrophobicity but also an inherent property of the sHsp that decides whether to bind stably or transiently.

Bacterial sHsps, similarly to their eukaryotic homologues, are considered to bind aggregation-prone polypeptides *via* the *N*-terminus, which is uncovered by a thermal dissociation of sHsp oligomers (Strozecka et al., 2012; Altenhoff et al., 2013; Chernova et al., 2020), and hydrophobic patches of the α-crystallin domain (Fu et al., 2013). Intermediate sHsp-polypeptide complexes may later associate into bigger assemblies comprised of both unfolded substrates and multiple sHsps. These constitute a safe-storage for clusters of folding intermediates that are protected from further aggregation by an sHsp outer shell (Zwirowski et al., 2017). sHsps interaction with unfolding substrate not only protects the substrate from further aggregation but also preserves the substrate secondary structure (Ungelenk et al., 2016). However, it was only shown using yeast sHsps and the analogous activity for bacterial sHsps has to be confirmed.

*In vitro* work has revealed that assemblies built of sHsps and substrates are substantially smaller than substrate amorphous aggregates formed in the same conditions in the absence of sHsps (Chang et al., 1996; Ratajczak et al., 2009; Obuchowski et al., 2019). As a consequence, the surface to mass ratio for the assemblies is much bigger, which generates more sites at which the disaggregation and substrate refolding may potentially start. However, it is not known if *in vivo* association of sHsps with denatured substrates increases the surface to mass ratio, as observed *in vitro*.

In addition to the classical chaperone activity towards proteins, some sHsps were found to participate in membrane maintenance in *Synechocystis PCC 6803* and *Oenococcus oeni* (Horvath et al., 1998; Torok et al., 2001; Maitre et al., 2014). Analogically, this activity is exerted by dissociated species that bind to the bacterial inner membrane, reducing its fluidity in stress conditions or in the presence of organic solvents (Torok et al., 2001; Capozzi et al., 2011; Maitre et al., 2012). sHsps were also found to stabilize thylakoid membranes in photosynthesizing cyanobacteria (Nakamoto and Honma 2006) or be involved in membrane fluidification in *Lactobacillus plantarum*, contributing to its cryotolerance (Arena et al., 2019). Despite the substrate difference, these activities seem similar to the classical chaperone protective activity and therefore the proteins exerting such activities (sHsps) were named lipochaperones (Maitre et al., 2014).

## Small Heat Shock Proteins Cooperate Functionally With Hsp70 and Hsp100 Chaperones in Refolding of the Aggregated Substrate

The introduction of sHsps to the family of molecular chaperones has raised fundamental questions regarding their possible relations to other, ATP-dependent chaperones in orchestrating cellular proteostasis. This drove the research on sHsps toward more precise integration in the network of molecular chaperones and their interactions. As described in the previous section, sHsps were shown to create sHsps-substrate assemblies upon aggregation initiation, which provoked the obvious concerns about the later fate of these structures.

**FIGURE 2:**
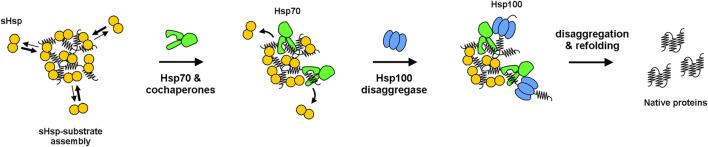
Model of refolding of substrates from sHsp–substrate assemblies by Hsp100-Hsp70 bi-chaperone system. sHsps dynamically bind and dissociate from the assemblies, competing with Hsp70 for binding sites. Binding of Hsp70 to assemblies allows for Hsp100 (ClpB) disaggregase recruitment, which initiates substrate disaggregation and refolding.

There are two possible scenarios for protein aggregates—either degradation by proteases or disaggregation and refolding. The second is mediated by Hsp70 system (DnaK, DnaJ and GrpE cochaperones in bacteria) either cooperating with Hsp100 disaggregase (ClpB in bacteria) in some organisms or acting alone in others (e.g. in metazoans). The very first connection between the latter scenario and bacterial sHsps was provided by Veinger and colleagues (Veinger et al., 1998), who *in vitro* explored disaggregation and refolding mediated by *E. coli* DnaK-ClpB bi-chaperone system in the presence or absence of IbpB (Figure 2). While the ability of sHsps to form assemblies was already known, the authors aimed to investigate whether IbpB binding has an impact on substrate disaggregation. They showed that IbpB does indeed influence the later refolding, facilitating it when present upon the denaturation step. Together with similar studies on an eukaryotic sHsp (Ehrnsperger et al., 1997), it seeded the hypothesis, that sHsps stabilize folding intermediates into assemblies that constitute a reservoir for their subsequent refolding. Later, yet another link between sHsps and 'big' chaperones was provided by Mogk et al. (2003b). They showed that *E. coli* sHsps, IbpA and IbpB, cooperate with ClpB and the DnaK system *in vitro* and *in vivo* and that IbpA and IbpB become essential for cell viability when DnaK levels are reduced (Mogk et al., 2003a).

Intuitively, one could think that such a refolding reservoir – as it was shown for *E. coli* IbpB (Veinger et al., 1998) - should generally facilitate disaggregation by Hsp70-Hsp100 bi-chaperone system. Indeed, it was reported that the denaturation of several different substrates in the presence of sHsps substantially increases the subsequent ClpB-DnaK-dependent refolding efficiency (Matuszewska et al., 2005; Mogk et al., 2003a; Mogk et al., 2003b). However, it was also noticed that it is not universal. *E. coli* IbpA protein, which efficiently forms assemblies, was reported to possess an evident inhibitory activity towards disaggregation and refolding in the absence of its IbpB paralog (Matuszewska et al., 2005; Ratajczak et al., 2009). This Janus-faced behavior of sHsps was not really explained until 2017, when Zwirowski and colleagues (Zwirowski et al., 2017) proposed a model for the interplay between sHsps and Hsp70 system in the refolding of aggregated substrates. They showed that the sHsp-induced inhibition is observed solely at low Hsp70 concentration and above a certain Hsp70 threshold sHsp presence in aggregates provides a substantial boost in disaggregation. This led to several mechanistic experiments, where the authors showed that the long-pursued sHsp interaction with other chaperones is, in fact, indirect. It is based on a simple Hsp70-sHsp competition for substrate polypeptides. Only the Hsp70 molecules that win the competition and bind the aggregate may further recruit, dock and stimulate the Hsp100 disaggregase for a polypeptide extraction (Rosenzweig et al., 2013; Miot et al., 2011; Liberek et al., 2008; Mogk et al., 2015) (Figure 2). The Hsp70-dependent release of sHsp from aggregates formed in stressed cells was also previously shown in cyanobacteria (Basha et al., 2004).

Given that sHsps have to effectively bind misfolding peptides and swiftly release them upon the Hsp70 action, a serious evolutionary trade-off has emerged. One sHsp simply cannot be a stable binder to form assemblies and, at the same time, promote disaggregation. Although most bacteria utilize just one sHsp, there are species expressing more of them (Haslbeck et al., 2005). Recently, Obuchowski et al. (2019) have shown that species from *Enterobacterales* clade have evolved an sHsp system of two cooperating components. One is a canonical IbpA that is a tight binder that is hard to outcompete from the substrate by Hsp70, and the other one, IbpB, is unable to stably bind the substrate and, therefore, can hardly modulate polypeptide aggregation (Ratajczak et al., 2009; Obuchowski et al., 2019). Such observations about the properties of these two sHsps come not only from *in vitro* experiments but also from *in vivo* studies which showed that IbpA is present exclusively in the aggregated protein fraction, while IbpB in the absence of IbpA is found mostly in the cytosolic soluble fraction (Kuczynska-Wisnik et al., 2002). Acting as a complex, they can both efficiently scavenge unfolding polypeptides and be removed from assemblies upon Hsp70 binding (Obuchowski et al., 2019). However, it is worth noting that using this data to induce conclusions about non-*Enterobacterales* should be done with great care, as it would require an assumption of convergence. It was already shown not to be the case for *D. radiodurans*, also expressing two paralogous sHsps that do not cooperate with each other, at least not in counteracting aggregation (Bepperling et al., 2012).

As already noted, different bacteria may contain varying numbers of sHsps. Known examples include species with only single sHsp, such as *Erwinia amylovora* or *Vibrio harveyi* (Klein et al., 2001; Obuchowski et al., 2019), two sHsps, like *E. coli* or *D. radiodurans* (Bepperling et al., 2012; Obuchowski et al., 2019) as well as three sHsps, like *L. plantarum* (Arena et al., 2019) or *Pseudomonas putida* (Krajewski et al., 2014). There are also more extreme cases like rhizobia, which possess large superfamily of sHsp, grouped in two distinct classes. The best studied example, *Bradyrhizobium japonicum*, contains seven identified sHsp genes as well as at least five more sHsps indicated by proteomic analysis (Münchbach et al., 1999). Studied examples of sHsps from bacteria expressing single and multiple sHsps revealed that their general principles of function are somewhat similar to sHsps from *E. coli*. sHsps from both single (Klein et al., 2001; Obuchowski et al., 2019) and multi-protein sHsp systems (Studer and Narberhaus 2000; Krajewski et al., 2014) form potentially mixed (in case of multi-protein systems) oligomers and interact with substrate proteins when the temperature rises (Studer and Narberhaus 2000; Klein et al., 2001; Obuchowski et al., 2019). Still, to date knowledge on bacterial sHsps would benefit from in depth analysis of sHsp-substrates complexes, both in terms of formation kinetics and structural organization. The spectrum of sHsps protein substrates at stress conditions is also hardly defined.

## Conclusion

Summing up, although different bacteria possess a different number of sHsp genes of limited conservation, all bacterial sHsps have consensus features defining their general activity. Most of all, it is the ATP-independent ability to bind the substrate following heat activation. sHsps bind substrates either stably, storing polypeptides for subsequent Hsp100-Hsp70 disaggregating machinery action, or transiently for unfolding prevention. Both activities positively influence protein homeostasis, increasing bacterial capabilities to survive stress conditions. These activities are always orchestrated by the very same, strikingly conserved structure of α-crystallin domain and flanking termini - showing that for this purpose it is a highly optimal solution that was provided very early in the evolution of chaperone systems.
